# Alternation of the Autonomic Nervous System Is Associated With Pulmonary Sequelae in Patients With COVID-19 After Six Months of Discharge

**DOI:** 10.3389/fphys.2021.805925

**Published:** 2022-01-21

**Authors:** Tao Bai, Dan Zhou, Feierkaiti Yushanjiang, Dongke Wang, Dongmei Zhang, Xinghuang Liu, Jun Song, Jianchu Zhang, Xiaohua Hou, Yanling Ma

**Affiliations:** ^1^Division of Gastroenterology, Union Hospital, Tongji Medical College, Huazhong University of Science and Technology, Wuhan, China; ^2^Xinjiang Medical University, Ürümqi, China; ^3^Key Laboratory of Pulmonary Diseases of Health Ministry, Department of Respiratory and Critical Care Medicine, Union Hospital, Tongji Medical College, Huazhong University of Science and Technology, Wuhan, China

**Keywords:** COVID-19, pulmonary sequelae, heart rate variability, pulmonary diffusion dysfunction, autonomic nervous system

## Abstract

Previous studies suggest that autonomic dysfunction is associated with disease severity in acute phase in patients with coronavirus disease 2019 (COVID-19). However, the association between autonomic dysfunction and pulmonary sequelae in patients with COVID-19 is unknown. We conducted a prospective study to investigate the association between autonomic dysfunction and pulmonary sequelae in patients with COVID-19 discharged for 6 months. We included 40 eligible participants and collected the following indicators: heart rate variability (HRV), pulmonary function tests (PFTs), lung X-ray computed tomography (CT), routine blood parameters, liver function parameters, and lymphocyte subsets. We found that at 6 months post-discharge, HRV still had a tight correlation with pulmonary fibrosis. There was a significant difference in HRV between patients with and without diffusion dysfunction, but HRV did not differ between patients with or without ventilatory dysfunction. Diffusion dysfunction and pulmonary fibrosis were tightly associated, and HRV index changes in patients with diffusion dysfunction had the same trend as that of patients with pulmonary fibrosis. They had a lower standard deviation of NN intervals (SDNN), the standard deviation of the average NN intervals (SDANN), and the triangular index, but a higher ratio between LF and HF power (LF/HF). In addition, WBC, neutrophils, and CD4/CD8 were correlated with pulmonary fibrosis and HRV. We concluded that autonomic dysfunction is closely associated with pulmonary fibrosis and diffusion dysfunction, and immune mechanisms may potentially contribute to this process.

## Introduction

According to the Coronavirus Resource Center at Johns Hopkins University, the global pandemic caused by coronavirus disease 2019 (COVID-19) has affected more than 230 million people. There have been more than 4.4 million deaths, and 180 million people have recovered (Lewis et al., 2021; Safont et al., 2021). With such a significant recovered population, we must be concerned about the long-term lung damage caused by COVID-19 infection (Zhao et al., 2020). The main pulmonary sequelae in patients with COVID-19 after discharge are pulmonary fibrosis and diffusion dysfunction, but mechanisms are unclear (Huang et al., 2021). Pulmonary fibrosis can seriously affect the quality of life of patients and impose a heavy financial burden on them (Lee et al., 2020; Leung et al., 2020). Its median survival is 3–5 years after diagnosis and is highly correlated with treatment (Meyer, 2017). So, early identification and intervention of pulmonary fibrosis are essential to improve the quality of survival. Heart rate variability (HRV) is the most valuable non-invasive test to assess the function of the autonomic nervous system (ANS; Ahmad et al., 2009). Some researchers found that ANS dysfunction appeared early in patients with COVID-19. In addition, even under the influence of factors, such as hypoxia and stress, HRV was still associated with the severity of patients in the acute phase (Natarajan et al., 2020; Luong et al., 2021). Severe patients without improved HRV needed longer to clear the virus and recover, indicating that HRV can be used as a non-invasive predictor for short-term clinical outcomes (Pan et al., 2021). Meanwhile, some studies suggest that the immune system is associated with long-term autonomic disorders and pulmonary sequelae in patients with COVID-19 (Kenney and Ganta, 2014; Hasty et al., 2021; Wang et al., 2020; Wu et al., 2021). However, we do not know whether there are associations between long-term autonomic disorders and pulmonary sequelae (Wang et al., 2020; Dani et al., 2021; McDonald, 2021).

In this study, we aimed to discuss the association between ANS dysfunction and pulmonary fibrosis sequelae. All the post-discharge patients were within normal limits for all physiological indicators, so HRV can more accurately and credible evaluate the function of the ANS.

## Materials and Methods

### Study Design and Participants

In this prospective study, we included 40 patients who had been hospitalized at the Union Hospital, Tongji Medical College, Huazhong University of Science and Technology between January 10 and February 10, 2020, for COVID-19 infection, and data were counted at 6 months after their discharge from the hospital. Demographics, clinical characteristics, pulmonary function tests, lung X-ray computed tomography (CT), and HRV were collected in the included patients. The study was approved by the Ethics Committee of the United Hospital. All patients included in this prospective study provided written informed consent at the time of admission.

### Demographics and Clinical Data

We recorded demographic characteristics [e.g., age, gender, and body mass index (BMI)] and detailed clinical data, such as post-discharge clinical symptoms (e.g., fever, cough, and diarrhea), routine blood parameters [e.g., white blood cell (WBC), red blood cell (RBC), and the mean corpuscular hemoglobin concentration (MCHC)], liver function parameter (e.g., aspartate transaminase (AST), alanine transaminase (ALT), and AST/ALT) were assessed in all patients. We invited all patients to participate in the immune system examination, but only 24 participants agreed to complete, such as lymphocyte subsets (e.g., CD4, CD8, and CD4/CD8). Our method of detecting lymphocyte subsets is using flow cytometry, the flow cytometer we used was the FACS Canto (BD, United States) and the reagent test kit used was the Lymphocytes Subgroup Typing Kit (Human) produced by BD Medical Devices (Shanghai) Co., Ltd. (Shanghai, China). The severity of illness, presence of acute respiratory distress syndrome (ARDS) in acute phase of COVID-19 and past medical history of each participant were retrieved from the electronic medical record of hospital. The diagnosis and disease severity of COVID-19 of all patients were based on the *New Coronavirus Pneumonia Diagnosis and Treatment Plan (Trial 7)* (Jing-Ya et al., 2020). The diagnosis of ARDS of all patients were based on *the American-European Consensus Conference on ARDS* (Fan et al., 2018). Past medical history includes: type 2 diabetes, hyperlipidemia, heart disease, and cardiopulmonary disease.

### Lung X-ray Computed Tomography

All 40 patients completed lung CT performed by the skilled operators and the examination reports were reviewed by clinically experienced physicians. None of the patients had any disease-causing pulmonary fibrosis or was taking medications that could cause pulmonary fibrosis prior to the COVID-19 infection. The lung CT features of patients with a confirmed diagnosis of pulmonary fibrosis on examination report can be summarized as follows: ground glass opacity, fiber streak shadow, tractive bronchiectasis, reticulation, and bronchovascular bundle distortion (Thannickal et al., 2004; Meyer, 2017).

### Pulmonary Function Tests

We performed pulmonary function tests (PFTs) using dry spirometry in all 40 patients. To avoid measurement errors caused by the instruments, we had conducted a professional and rigorous inspection of all the devices used in the study. PFTs and diffusing capacity of the lung for carbon monoxide (DLCO) measurements were performed according to American Thoracic Society (ATS)/China primary guidelines for routine PFTs (Culver et al., 2017; National Health Commission of the People’s Reupblic of China, 2020). PFT parameters were expressed as absolute and percentage of a theoretical value calculated by Global Lung Function 2012 equations (Quanjer et al., 2012). The final measurement includes lung volume, ventilation, and diffusion function. According to the guide, the definition of ventilation dysfunction is that forced expiratory volume (FEV)1/forced vital capacity (FVC) < 70% and the definition of diffusion dysfunction is DLCO/80% DLCO pred < 1 (Quanjer et al., 2012; Culver et al., 2017; Dempsey and Scanlon, 2018). A professional technician reviewed the content to exclude obvious errors due to poor patient cooperation, etc. We will use only the report form data signed by both the clinician and technician.

### Heart Rate Variability Recording

Each patient wore a Holter detector to record ambulatory and continuous ECG data over 24 h. ECG data, such as time-period, duration, value, and type of arrhythmia, were fully checked and validated by experienced physicians. Intervals with significant variations were excluded to prevent measurement operation errors. The HRV data were calculated according to the time domain analysis method, which uses the ECG waveforms measured continuously to directly calculate and analyze the relationship between the time series of connected heartbeats. These include the standard deviation of NN intervals (SDNN; normal value: 141 ± 39 ms; reflects total HRV); standard deviation of the average NN intervals (SDANN; normal value: 127 ± 35 ms; reflects primarily circadian HRV); the square root of the mean of the sum of the squares of differences between adjacent NN intervals (RMSSD; normal values: 27 ± 12 ms; reflects vagal activity); percentage differences between adjacent NN intervals that are greater than 50 ms (pNN50; normal values: 16.7 ± 12.3%; reflects vagal activity). Frequency-domain analysis included low-frequency power 0.04–0.15 Hz (LF; normal values: 300–1,750 ms^2^; reflect combination of sympathetic nervous system (SNS) and peripheral nervous system (PNS) influences, captures baroreflex rhythms), high-frequency power 0.15–0.4 Hz (HF; normal values: 50–120 ms^2^; under normal circumstances reflects vagal activity), and the ratio of low- to high-frequency power (LF/HF; normal values: 1–3; reflect SNS/PNS balance) (Stein and Pu, 2012; Shaffer and Ginsberg, 2017; Mejia-Mejia et al., 2020; Pan et al., 2021).

### Statistical Analysis

All categorical variables were compared using the Mann–Whitney *U*-test or the Fisher’s exact test, and all continuous variables were compared using the *t*-test, the paired *t*-test, or the Wilcoxon signed-rank test, as appropriate. Categorical data are expressed as proportions (%). Continuous data were expressed as mean. Pearson’s rank correlation and Spearman’s rank correlation analysis were used for correlation analysis. Receiver operating characteristic (ROC) plots were generated evaluating four HRV indexes in predicting the pulmonary fibrosis. All statistical analyses were processed using SPSS 26.0 (IBM, Chicago, IL, United States) and R (Version 4.1.1). The *P*-value < 0.05 was considered significant.

## Results

### Demographic and Clinical Characteristics of Participants

We included 40 patients with COVID-19 after 6 months of discharge: the mean age was 55.1 ± 13.9 years, and 23 (68%) were female. The mean BMI was 25.4 ± 2.5 kg/m^2^. In the acute phase, 14 (35%) of the 40 participants were classified as mild patients and 26 (65%) as severe patients. Older individuals were predominantly clinically typed as severe (*P* < 0.05). The most common symptom after discharge for 6 months was cough (30%, 12/40), followed by muscle pain (25%, 10/40), diarrhea (20%, 8/40), and constipation (20%, 8/40). There were 21 (52.5%, 21/40) patients who had ARDS in the acute phase, 2 (5%) patients had a drinking history, 7 (17.5%) had a smoking history. About medical history, there were 5 (12.5%) patients that had the cardiopulmonary disease, 14 (35%) of them had heart disease, 12 (30%) had hyperlipidemia, and 6 (15%) had type 2 diabetes (Tables 1.1–1.3).

**TABLE 1.1:** Clinical characteristics of all patients and grouped by ventilatory dysfunction.

Variable	Total	No ventilatory dysfunction	With ventilation dysfunction	*P* value
	(*N* = 40)	(*N* = 33)	(*N* = 7)	
Sex (male), N (%)	17 (42.5%)	15 (45.5%)	2 (28.6%)	0.577
Age, Mean, year	55.1 ± 13.9	53.1 ± 13.5	64.1 ± 11.8	0.162
BMI	25.4 ± 2.5	25.7 ± 2.6	24 ± 0.4	0.807
Clinical typing of severe and above	14 (35%)	10 (30.3%)	4 (57%)	0.401
Have ARDS	21 (52.5%)	20 (60.6%)	1 (14.3%)	0.754
Drinking history	2 (5%)	2 (6.1%)	0 (0%)	0.058
Smoking history	7 (17.5%)	7 (21.1%)	0 (0%)	0.137
History of cardiopulmonary disease	5 (12.5%)	4 (12.1%)	1 (14.3%)	0.945
History of heart disease	14 (35%)	12 (36.3%)	2 (28.5%)	0.754
History of hyperlipidemia	12 (30%)	7 (21.2%)	5 (71.4%)	0.037
History of Type 2 diabetes	6 (15%)	4 (12.1%)	2 (28.5%)	0.507
**Post-discharge symptoms and signs, n (%)**				
Fever	1 (2.5%)	1 (3%)	0 (0%)	0.937
Cough	12 (30%)	12 (36.3%)	0 (0%)	0.228
Breathing difficulties	17 (42.5%)	12 (36.4%)	4 (57.1%)	0.425
Diarrhea	8 (20%)	6 (18.1%)	2 (28.6%)	0.425
Muscle pain	10 (25%)	9 (27.9%)	1 (14.3%)	0.843
Pulmonary fibrosis, n (%)	23 (57.5%)	16 (48.5%)	7 (100%)	0.107
**CT findings (%)**				
Ground glass	–	–	2 (28.6%)	–
Opacity fiber	–	–	7 (100%)	–
Streak shadow	–	–	2 (28.6%)	–
Tractive bronchiectasis reticulation	–	–	2 (28.6%)	–
Bronchovascular bundle distortion	–	–	1 (14.3%)	–

### Lung Function and Lung X-ray Computed Tomography Appearances of Post-discharge Participants

First, there were seven patients (17.5%) who had ventilation dysfunction (Table 1.1). The mean age of the ventilation disorder patients was 64.1 ± 11.8 years, which was not significantly correlated with the ventilation dysfunction (*P* = 0.162). The correlations between ventilation disorders and the severity of disease, ARDS, a history of smoking, and a history of alcohol consumption were not significant (*P* > 0.05). However, a history of hyperlipidemia was correlated with pulmonary ventilation dysfunction (*P* = 0.037). Pulmonary ventilation dysfunction did not associate significantly with pulmonary fibrosis, and only 7 of 23 patients with pulmonary fibrosis sequelae developed pulmonary ventilation dysfunction (*P* = 0.107).

Second, 22 patients (55.0%) had diffusion disorder with a mean age of 62.0 ± 9.7 years (*P* = 0.001), being significant (Table 1.2). There were more women patients with diffusion dysfunction (*p* = 0.039). The differences between BMI, clinical typing of severe, ARDS, and past medical history were not significant (*p* > 0.05). Especially, in patients with diffusion dysfunction, breathing difficulties and cough are more apparent symptoms after 6 months of discharge. Importantly, 22 of 23 patients with COVID-19 after 6 months discharge who had pulmonary fibrosis had pulmonary diffusion dysfunction, and there was a tight correlation between pulmonary fibrosis and pulmonary diffusion dysfunction (*P* < 0.01).

**TABLE 1.2:** Clinical characteristics of all patients and grouped by diffusion dysfunction.

Variable	Total	No diffusion dysfunction	With diffusion dysfunction	*P* value
	(*N* = 40)	(*N* = 18)	(*N* = 22)	
Sex (male), N (%)	17 (42.5%)	14 (77.8%)	3 (13.7%)	0.039
Age, Mean, year	55.1 ± 13.9	46.6 ± 13.2	62.0 ± 9.7	0.001
BMI	25.4 ± 2.5	25.7 ± 2.1	24.8 ± 2.5	0.299
Clinical typing of severe and above	26 (65%)	10 (55.6%)	16 (72.7%)	0.352
Have ARDS	21 (52.5%)	8 (44.4%)	13 (59%)	0.443
Drinking history	2 (5%)	2 (11.1%)	0 (0%)	0.563
Smoking history	7 (17.5%)	5 (27.8%)	2 (9.1%)	0.325
History of cardiopulmonary disease	5 (12.5%)	3 (16.7%)	2 (9.1%)	0.904
History of heart disease	14 (35%)	4 (22.2%)	10 (45.5%)	0.219
History of hyperlipidemia	12 (30%)	3 (16.7%)	9 (40.1%)	0.199
History of type 2 diabetes	6 (15%)	5 (27.8%)	1 (4.5%)	0.366
**Post-discharge symptoms and signs, n (%)**				
Fever	1 (2.5%)	0 (0%)	1 (4.3%)	0.829
Cough	12 (30%)	5 (27.8%)	7 (31.8%)	0.626
Breathing difficulties	16 (40%)	5 (27.8%)	11 (50%)	0.665
Diarrhea	8 (20%)	3 (16.6%)	5 (22.7%)	0.745
Muscle pain	10 (25%)	4 (22.2%)	6 (27.2%)	0.685
Pulmonary fibrosis, n (%)	23 (57.5%)	3 (16.7%)	20 (90.1%)	*P* < 0.01
**CT findings (%)**				
Ground glass	–	–	7 (31.8%)	–
Opacity fiber	–	–	19 (86.3%)	–
Streak shadow	–	–	7 (31.8%)	–
Tractive bronchiectasis reticulation	–	–	9 (40.1%)	–
Bronchovascular bundle distortion	–	–	8 (36.3%)	–

Finally, pulmonary fibrosis was present in 23 of the 40 participants (57.5%) (Table 1.3). The mean age of patients with pulmonary fibrosis was 59.7 ± 12.5 years, which was higher than patients without pulmonary fibrosis (*P* = 0.018). The correlation between pulmonary fibrosis and the clinical type of patients, presence of ARDS, history of smoking, history of alcohol consumption, and history of underlying diseases was not significant (*P* > 0.05).

**TABLE 1.3:** Clinical characteristics of all patients and grouped by pulmonary fibrosis.

Variable	Total	No pulmonary fibrosis	With pulmonary fibrosis	*P* value
	(*N* = 40)	(*N* = 17)	(*N* = 23)	
Sex (male), N (%)	17 (42.5%)	14 (82.4%)	*N* = 3 (13.0%)	0.432
Age, Mean, year	55.1 ± 13.9	54.1 ± 12.9	59.7 ± 12.5	0.018
BMI	25.4 ± 2.5	25.7 ± 2.1	25.4 ± 2.7	0.432
Clinical typing of severe and above	26 (65%)	10 (55.6%)	16 (72.7%)	0.277
Have ARDS	21 (52.5%)	8 (44.4%)	13 (59%)	0.626
Drinking history	2 (5%)	2 (11.1%)	0 (0%)	0.533
Smoking history	7 (17.5%)	5 (27.8%)	2 (9.1%)	0.588
History of cardiopulmonary disease	5 (12.5%)	3 (16.7%)	2 (9.1%)	0.957
History of heart disease	14 (35%)	4 (22.2%)	10 (45.5%)	0.165
History of hyperlipidemia	12 (30%)	3 (16.7%)	9 (40.1%)	0.090
History of type 2 diabetes	6 (15%)	5 (27.8%)	1 (4.5%)	0.401
**Post-discharge symptoms and signs, n (%)**				
Fever	1 (2.5%)	0 (0%)	1 (4.3%)	0.829
Cough	12 (30%)	5 (27.8%)	7 (31.8%)	0.957
Breathing difficulties	17 (42.5%)	4 (23.5%)	13 (56.5%)	0.329
Diarrhea	8 (20%)	3 (16.6%)	5 (22.7%)	0.829
Muscle pain	10 (25%)	4 (22.2%)	6 (27.2%)	0.134
**CT findings (%)**				
Ground glass	–^&^	–	7 (30.4%)	–
Opacity fiber	–	–	20 (86.9%)	–
Streak shadow	–	–	4 (17.3%)	–
Tractive bronchiectasis reticulation	–	–	9 (39.1%)	–
Bronchovascular bundle distortion	–	–	3 (13.1%)	–

*&: Data were not available.*

**FIGURE 1:**
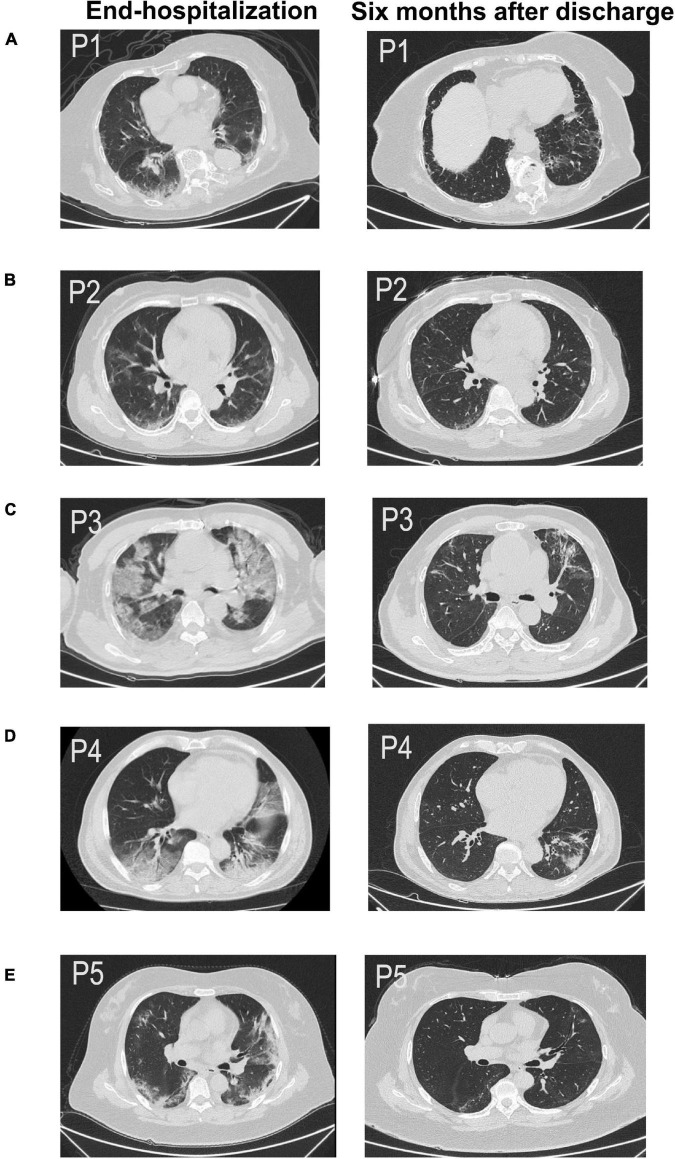
Chest CT scan of five patients **(A–E)** across two time periods, such as end-hospitalization and after 6 months discharge. All of them were pulmonary sequelae after 6 months of discharge (P1–P5). **(A)** CT image of a 51-year-old woman (P1) showing fiber streak shadow, reticulation, and tractive bronchiectasis 6 months after discharge. **(B)** CT image of a 57-year-old woman (P2) showing fiber streak shadow, reticulation, and tractive bronchiectasis 6 months after discharge. **(C)** CT image of a 76-year-old man (P3) showing ground glass opacity, fiber streak shadow, reticulation, and tractive bronchiectasis 6 months after discharge. **(D)** CT image of a 63-year-old man (P4) showing fiber streak shadow, reticulation, and bronchovascular bundle distortion. **(E)** CT image of a 65-year-old woman (P5) showing ground glass opacity, fiber streak shadow, and reticulation.

However, we observed the CT findings of all patients with pulmonary dysfunction and found that almost all patients with diffusion dysfunction had fiber streak shadow, reticulation, ground glass opacity tractive bronchiectasis, bronchovascular bundle distortion, and other typical manifestations of pulmonary fibrosis (Figure 1): fiber streak shadow (86.3%, 19/22), reticulation (40.1%, 9/22), bronchovascular bundle distortion (36.3%, 8/22), ground glass opacity (31.8%, 7/22), and tractive bronchiectasis (31.8%, 7/22) (Table 1.2). Contrary to patients with diffusion dysfunction, the patients with ventilation dysfunction had fewer fibrosis features on CT (Table 1.1).

### The Correlation Among Pulmonary Fibrosis, Diffusion Dysfunction, and Heart Rate Variability

**FIGURE 2:**
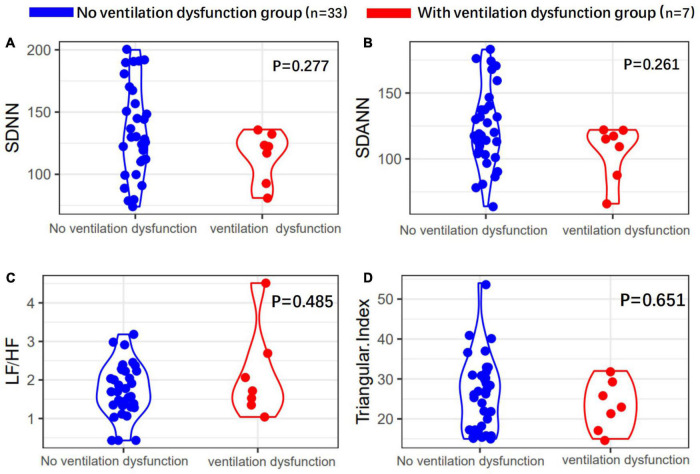
Heart rate variability (HRV) measurements in with or without ventilation dysfunction patients’ group. **(A)** SDNN, **(B)** SDANN, **(C)** LF/HF, and **(D)** triangular index are shown.

**FIGURE 3:**
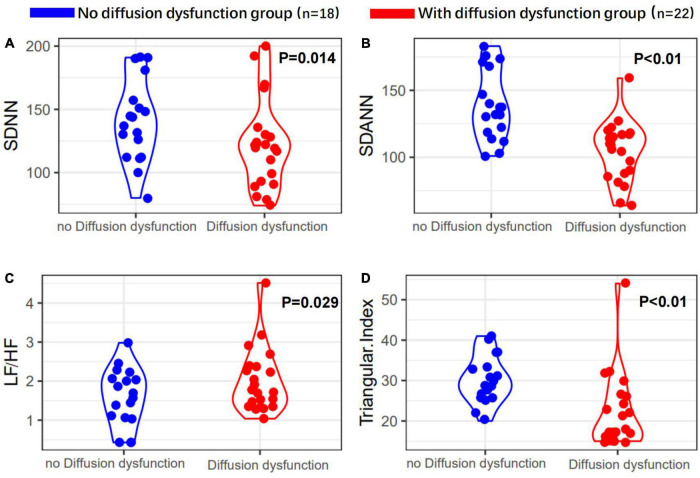
Heart rate variability measurements in with or without diffusion dysfunction patients’ group. **(A)** SDNN, **(B)** SDANN, **(C)** LF/HF, and **(D)** triangular index are shown.

**FIGURE 4:**
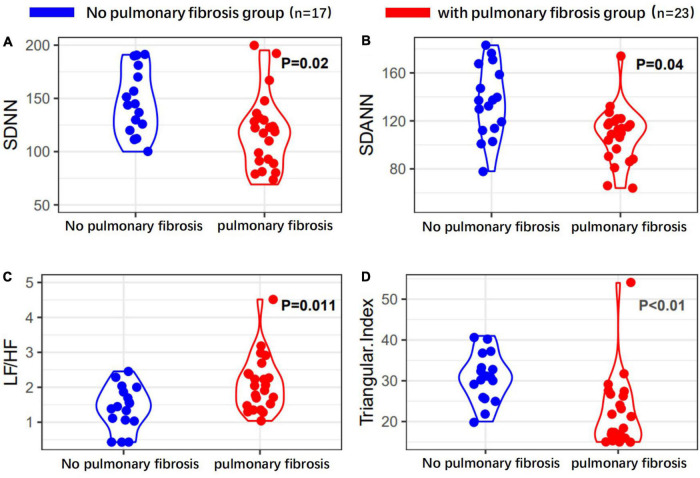
Heart rate variability measurements in with or without pulmonary fibrosis patients’ group. **(A)** SDNN, **(B)** SDANN, **(C)** LF/HF, and **(D)** triangular index are shown.

**FIGURE 5:**
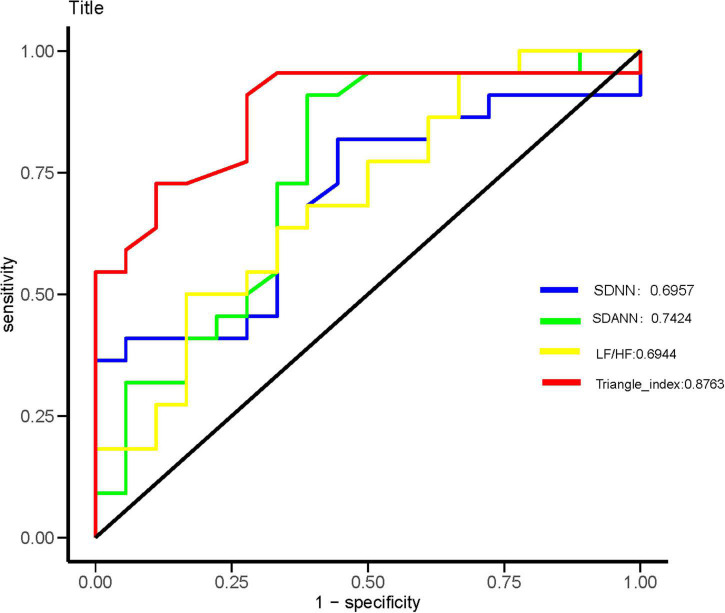
Receiver operating characteristic (ROC) analysis for significant HRV variables. The sensitivity and specificity of SDNN, SDANN, LF/HF, and triangular index for the severity of coronavirus disease 2019 (COVID-19).

Heart rate variability indices were tightly correlated with diffusion dysfunction and pulmonary fibrosis (*P* < 0.05) (Figures 2–4). SDNN, SDANN, and triangular index were lower in the patients with diffusion dysfunction (*P* = 0.014, *P* < 0.01, and *P* < 0.01), while LF/HF was higher (*P* = 0.029). The patients with pulmonary fibrosis showed the same trend as the patients with diffusion dysfunction, with lower SDNN, SDANN, triangular index, and higher LF/HF than the non-fibrosis patients (*P* = 0.02, *P* = 0.04, *P* < 0.01, and *P* = 0.011, respectively). All *P* values were calculated excluding BMI, age, weight, and other confounding factors. To assess the diagnostic value of the SDNN, SDANN, LF/HF, and triangular index, the ROC curve analysis was the best choice. The curves of all four indicators are located above the diagonal line, indicating that all have good sensitivity and specificity. The AUC of SDNN, SDANN, LF/HF, and triangular index for pulmonary fibrosis were 0.6957, 0.7424, 0.6944, and 0.8763, respectively (Figure 5).

**FIGURE 6:**
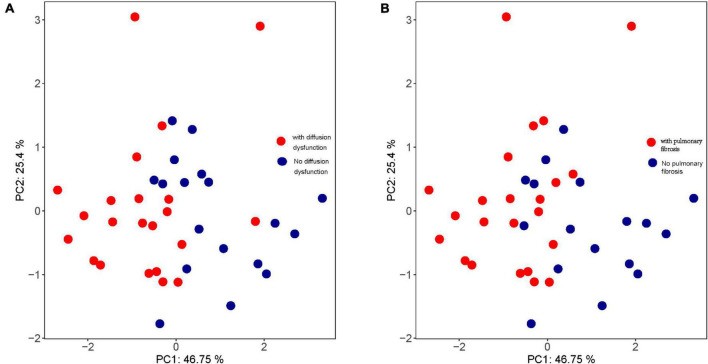
Principal component analysis (PCA) of **(A)** pulmonary diffusion dysfunction and **(B)** pulmonary fibrosis.

Moreover, the trend of HRV index changes in patients with diffusion dysfunction had the same trend as that of patients with pulmonary fibrosis (Figure 6). These results suggested that pulmonary fibrosis and diffusion dysfunction were also tightly associated.

### The Correlation Between Heart Rate Variability and Immune System

**FIGURE 7:**
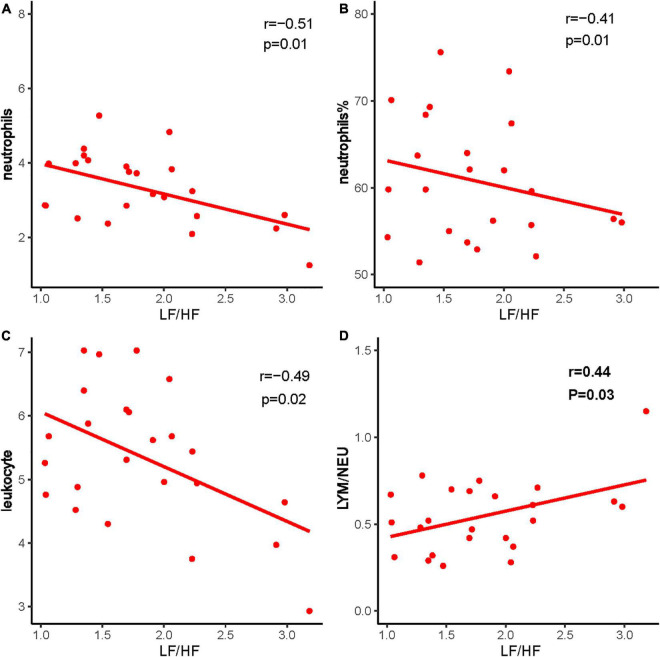
The correlation between LF/HF and immune system indexes. **(A)** LF/HF and neutrophils, **(B)** LF/HF and neutrophils %, **(C)** LF/HF and leukocyte, and **(D)** LF/HF and LYM/NEU. Red dots and red lines represents a patient with both HRV indicators and immune system related indicators.

**FIGURE 8:**
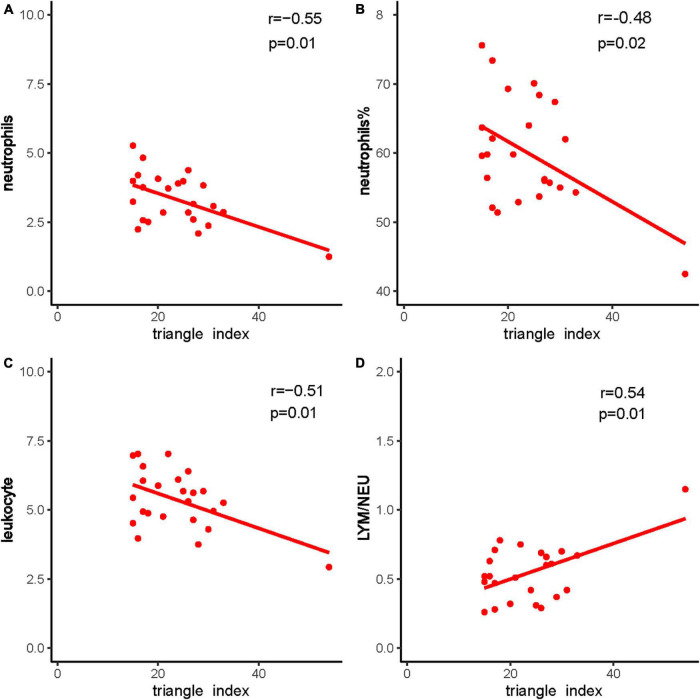
The correlation between triangle index and immune system indexes. **(A)** Triangle index and neutrophils, **(B)** triangle index and neutrophils %, **(C)** triangle index and leukocyte, and **(D)** triangle index and LYM/NEU. Red dots and red lines represents a patient with both HRV indicators and immune system related indicators.

**FIGURE 9:**
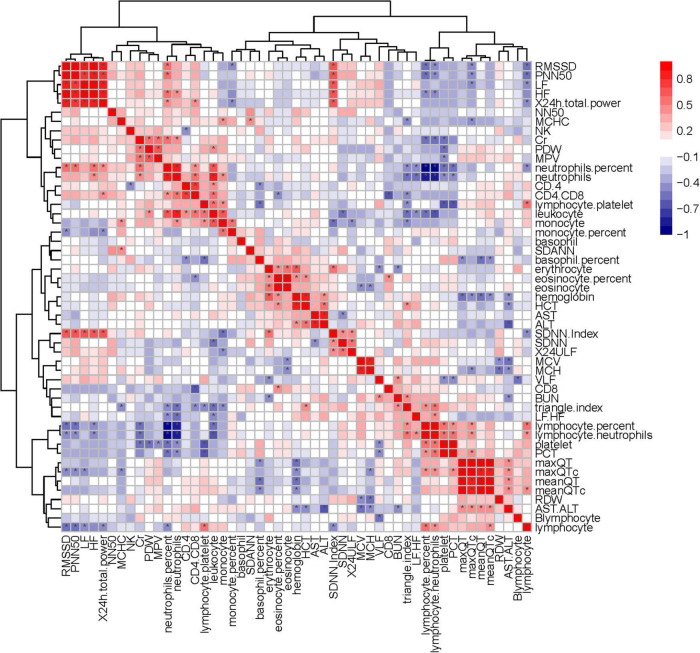
Correlation heatmap exhibiting the relationship among routine blood parameters, liver function parameters, lymphocyte subsets, and HRV indexes in all recruited patients with COVID-19 (the asterisk means *P* < 0.05).

Heart rate variability was correlated with the immune system. Triangle index and LF/HF showed significant negative correlations with neutrophils (*P* = 0.005, *R* =−0.552; *P* = 0.010, *R* =−0.513), neutrophils % (*P* = 0.019, *R* =−0.476; *P* = 0.001, *R* =−0.593), leukocyte (*P* = 0.011, *R* =−0.511; *P* = 0.016, *R* =−0.487), and positive correlations with lymphocyte/neutrophils (*P* = 0.006, *R* = 0.543; *P* = 0.032, *R* = 0.438) (Figures 7, 8). Moreover, CD4/CD8 was only tightly correlated with the triangle index. However, the correlation between SDNN and SDANN with immune-related indicators was not significant, and liver function parameters had no significant correlation (Figure 9).

## Discussion

This study found that autonomic dysfunction was tightly correlated with pulmonary sequelae in patients with COVID-19 after 6 months of discharge. Compared with the acute phase, the ventilatory dysfunction of many patients was relieved after discharge, but more patients had pulmonary diffusion dysfunction and pulmonary fibrosis sequelae. We support that the long-term lung injury in patients with COVID-19 was dominated by diffusion dysfunction and that the structural sequelae were reflected in fibrosis. Previous studies have suggested that HRV was related to the condition of patients in the acute phase, and we found that HRV was associated with the patient condition even after discharge. Similar to the severe patients with COVID-19 in the acute phase, some HRV indexes in patients with pulmonary sequelae in the chronic phase were also decreased, such as SDNN, SDANN, and triangular index (Pan et al., 2021). Patients whose HRV did not return to normal may take longer to recover lung function. However, no previous study focuses on the association between autonomic dysfunction with the chronic phase of patients with COVID-19.

The ANS plays an essential and irreplaceable role in maintaining the balance of body (Ulrich-Lai and Herman, 2009). HRV is the fluctuation in the time interval between consecutive heartbeats, the measurement of which is a non-invasive method of assessing the autonomic status (Ponomarev et al., 2021). Studies have found that HRV changes in systemic infections and that monitoring HRV can improve the diagnosis and prognosis of infections (Barnaby et al., 2019). In addition, HRV can be considered as a possible predictive marker for the acute inflammatory response in patients with COVID-19 (Del Rio et al., 2020). The potential role of autonomic dysfunction may play a critical role in COVID-19 morbidity and mortality (Barizien et al., 2021). Most importantly, the latest research suggests that HRV can predict the clinical outcomes of patients in the acute phase. In addition, dysautonomia may explain the persistent symptoms observed in long duration patients with COVID-19, such as fatigue (Wiersinga et al., 2020). So, our research innovatively studied the association between HRV and pulmonary sequelae.

Our results suggest that there is a potential correlation between chronic autonomic disorder and pulmonary fibrosis and HRV can be used as a non-invasive predictor of clinical outcome in the patients with COVID-19 after acute phases. Endothelial barrier disruption, dysfunctional alveolar-capillary oxygen transmission, and impaired oxygen diffusion capacity are collectively characteristic features of patients with COVID-19 in the acute phase. However, due to these characteristics, patients in the acute phase were often in a state of hypoxia or stress and had a faster breathing rate. HRV indexes were temporarily affected by these factors (Baptista et al., 2020; Wiersinga et al., 2020; Pan et al., 2021). Compared with in-hospital, all patients included in our study had normal blood oxygen saturation and their psychological pressure had also been reduced a lot so that we can reduce at least two acute interference factors (hypoxia and stress) on HRV (Leung et al., 2020; Wang et al., 2020). Therefore, HRV can more genuinely reflect the function of the ANS. Additionally, we excluded the underlying disease, weight, gender, age, and other possible interfering factors in the calculation. In addition, we selected four credible, representative, and widely used indicators as the main research objects: SANN and SDANN are very representative of the overall changes in HRV, and the triangular index is for the overall assessment of HRV from a geometric perspective, LF/HF can reflect changes in autonomic nerve function, especially LF/HF which has been widely used in the evaluation of condition of patients with sleep disorders and depression (Boudreau et al., 2013; Sgoifo et al., 2015; Shaffer and Ginsberg, 2017). Therefore, our results can more accurately investigate the correlation between long-term lung damage caused by COVID-19 infection and autonomic dysfunction. In addition, our results agreed with previous follow-up studies that autonomic long-term lung injury in patients with COVID-19 is dominated by diffusion dysfunction and pulmonary fibrosis (Faverio et al., 2021; Huang et al., 2021; Shah et al., 2021).

The ANS is closely associated with many regulating mechanisms, such as the immune system (e.g., CD4/CD8, lymphocyte, and neutrophils) (Kenney and Ganta, 2014; Wu et al., 2021). Some researchers believe that the COVID-19 can affect the condition of patient by affecting the immune system and HRV also related to immune system changes (Ahmad et al., 2009; Chowdhury et al., 2020; Tahaghoghi-Hajghorbani et al., 2020; Hasty et al., 2021). Our result suggests that HRV was indeed correlated with some immune system indicators, such as CD4/CD8 was correlated with the triangular index (*P* < 0.05) (Figure 9). Furthermore, PFTs were correlated with some immune system indexes, such as neutrophils and monocyte (*P* < 0.05) (Supplementary Figure 1). Therefore, our study may provide evidence that the immune system was interfered with COVID-19 and offer new ideas for the treatment of pulmonary fibrosis.

**FIGURE 10:**
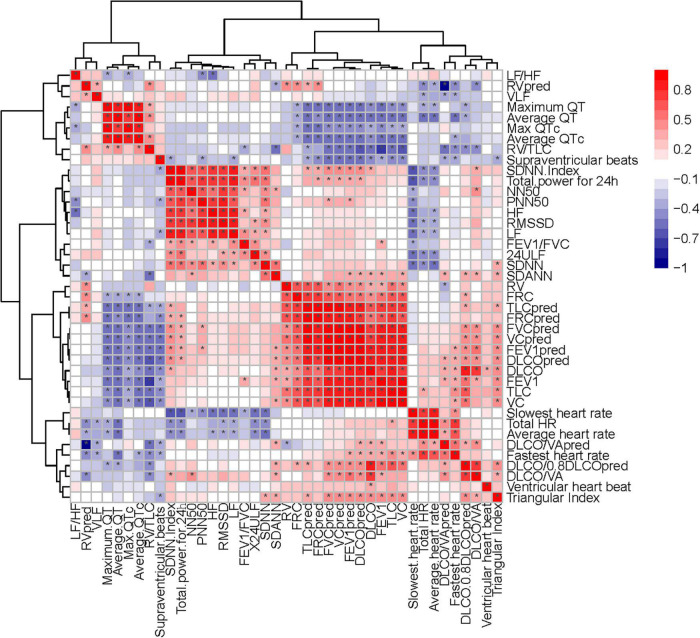
Correlation heatmap exhibiting the relationship among pulmonary function test, diffusing capacity of the lung for carbon monoxide (DLCO) related tests and HRV indexes in all recruited patients with COVID-19 (the asterisk means *P* < 0.05).

Heart rate variability-evaluation, as widely accepted and non-invasive, is cost-neutral and available for use under-study and clinical conditions (Xhyheri et al., 2012; Natarajan et al., 2020). In this study, we found that HRV was correlated with pulmonary fibrosis, pulmonary diffusion dysfunction, and immune system (Figures 9, 10). Thus, HRV should be considered when evaluating the therapeutic approaches of COVID-19. Patients with faster recovery from HRV often do not have pulmonary fibrosis. Not only pulmonary sequelae, previous studies indicated that immune system factors are inevitably linked to a well-balanced ANS (Czura and Tracey, 2005). Patients with more severe autonomic dysfunction will have a greater probability of pulmonary sequelae, but the development of pulmonary sequelae is not only affected by one factor. Long-term COVID-19 infected may lead to various immune system reactions, which can affect autonomic nerve activity (Wu et al., 2021). Direct viral invasion of neural parenchyma or *via* retrograde axonal transport could be a mechanism, too (Koralnik and Tyler, 2020). In some related diseases, the vagus nerve stimulation has been used as a part of the therapeutic approach (Baptista et al., 2020). Therefore, HRV indicators, immune related treatments, and vagus nerve stimulation (VNS) treatment methods can be considered in the process of rehabilitation of patients with COVID-19.

At present, the global research on the health of patients with COVID-19 after discharge is still in its infancy. The tight correlation between HRV and pulmonary fibrosis, lung function, immune system, and other symptoms after the acute phase is worthy of attention.

Our findings are preliminary, and our study is just based on a small sample of patients with COVID-19 in one hospital. If the sample size is large enough, there may be more in-depth discoveries. But the limitation does not affect the conclusion of the article, because based on the current data, it has been able to prove the correlation between the key indicators. To make the association between pulmonary fibrosis and HRV more valuable for clinical or research in the future, we could design a cohort study to discuss whether HRV can predict chronic patient outcomes.

## Conclusion

This study showed that autonomic dysfunction is significantly correlated with pulmonary diffusion dysfunction and pulmonary fibrosis after discharge among patients with COVID-19, and immune mechanisms may be correlated with the autonomic dysfunction and may play a potential role between the ANS and pulmonary sequelae.

## Data Availability Statement

The raw data supporting the conclusions of this article will be made available by the authors, without undue reservation.

## Ethics Statement

The studies involving human participants were reviewed and approved by the Research Ethics Commission of Wuhan Union Hospital. The patients/participants provided their written informed consent to participate in this study.

## Author Contributions

XH and YM developed the main idea of this study. TB developed the search strategy. DaZ independently completed the selection of studies, data extraction, assessment of risk of bias, and data synthesis. FY and DW arbitrated the disagreements and drafted and revised the original manuscript. All authors have read and approved the final manuscript.

## Conflict of Interest

The authors declare that the research was conducted in the absence of any commercial or financial relationships that could be construed as a potential conflict of interest.

## Publisher’s Note

All claims expressed in this article are solely those of the authors and do not necessarily represent those of their affiliated organizations, or those of the publisher, the editors and the reviewers. Any product that may be evaluated in this article, or claim that may be made by its manufacturer, is not guaranteed or endorsed by the publisher.

## Supplementary Material

Supplementary Figure 1 Correlation heatmap exhibiting the relationship between pulmonary function test, diffusing capacity of the lung for carbon monoxide (DLCO) related tests, immune system indexes and liver function tests in all recruited patients with coronavirus disease 2019 (COVID-19) (the asterisk means *P* < 0.05).
