# Supplementary figures and images for: Alternation of the Autonomic Nervous System Is Associated With Pulmonary Sequelae in Patients With COVID-19 After Six Months of Discharge
Source: Front Physiol. 2022 Jan 21;12:805925. doi: 10.3389/fphys.2021.805925 (PMC8814436; doi:10.3389/fphys.2021.805925)

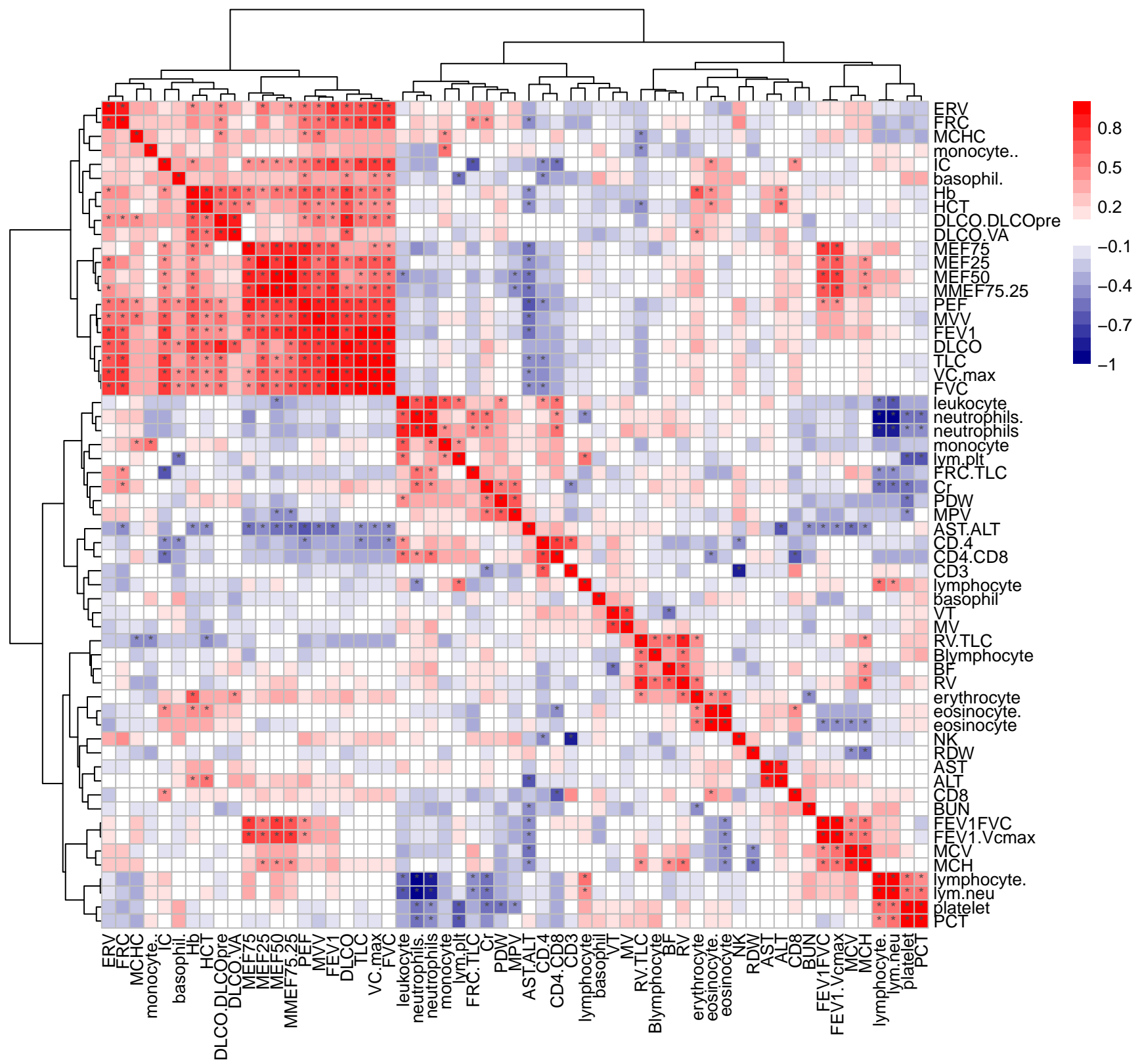

Supplement: Supplementary Figure 1 — Correlation heatmap exhibiting the relationship between pulmonary function test, diffusing capacity of the lung for carbon monoxide (DLCO) related tests, immune system indexes and liver function tests in all recruited patients with coronavirus disease 2019 (COVID-19) (the asterisk means P < 0.05). [file Data_Sheet_1.PDF]
